# Auditory processing neurons influence song evaluation and strength of mate preference in female songbirds

**DOI:** 10.3389/fncir.2022.994548

**Published:** 2022-10-03

**Authors:** Koedi S. Lawley, Thomas Fenn, Emily Person, Holly Huber, Kristina Zaharas, Perry Smith, Austin Coulter, Jonathan F. Prather

**Affiliations:** ^1^Department of Veterinary Integrative Biosciences, School of Veterinary Medicine and Biomedical Sciences, Texas A&M University, College Station, TX, United States; ^2^Neuroscience Program, Department of Zoology & Physiology, University of Wyoming, Laramie, WY, United States

**Keywords:** communication, perception - action, decision-making, neurobiology, hearing

## Abstract

Animals use a variety of complex signaling mechanisms to convey an array of information that can be detected by conspecifics and heterospecifics. Receivers of those signals perceive that information and use it to direct their subsequent actions. Thus, communication such as that which occurs between senders and receivers of vocal communication signals can be a powerful model in which to investigate the neural basis of sensory perception and action initiation that underlie decision-making. In this study, we investigated how female songbirds perceive the quality of acoustic signals (songs) performed by males and use that information to express preference for one song among many possible alternatives. We use behavioral measurement of song preference before and after lesion-induced alteration of activity in an auditory processing area (caudal nidopallium, NC) for which we have previously described its interconnections with other auditory areas and downstream reward pathways. Our findings reveal that inactivating NC does not change a female’s ability or willingness to perform behavioral indicators of mate choice, nor does it change their ability to identify the songs of individual males. However, lesioning NC does induce a decrease in the strength of song preference for specific males more than others. That decrease does not result in a complete elimination of preference, as female preferences for specific males are still evident but not as strongly expressed after lesioning of NC. Taken together, these data indicate that NC plays a role in a female’s strength of preference in song evaluation and mate choice, and activity in NC is an important facet of mate choice.

## Introduction

Organisms continuously perceive and process sensory information, determine the value of that information, and use it to select one action from many possible alternatives (Kable and Glimcher, 2009). These assessments and the associated actions result in decisions that have many biological and social impacts on the life of an organism. Such decisions can include where to forage, whether to flee, and perhaps the most evolutionarily significant decision of the partner with whom they will mate. In humans and other mammals, neural networks involving complex interactions between associational cortices and reward centers have been implicated in assessing stimulus value, and additional networks associated with motor initiation have also been well characterized (Gold and Shadlen, 2007). However, the neural mechanisms behind this complex processing of sensory information and the link between sensory information and motor activation remains poorly understood. Here we turn to an animal model to investigate those neural mechanisms and their associated roles.

Mate choice can be a useful model to explore the neural mechanisms of decision-making. Females of many species must perceive and evaluate sensory information such as plumage, ornamentation, behavioral displays, and vocalizations from members of the opposite sex to evaluate a male’s quality (reviewed in Catchpole and Slater, 2008). The female can then use that information to selectively engage in courtship and copulatory behaviors with the suitor that she prefers most strongly. In the present study, we investigated female songbirds to identify the neural structures through which females recognize and evaluate the quality of male songs and use that information to choose their mates.

The behaviors expressed in songbird courtship and the circuits of the songbird brain provide an experimentally approachable context to characterize the links between neural activation and behavior (reviewed in Murphy et al., 2020; Fujii et al., 2022). In many songbird species, including the Bengalese finches (BF; *Lonchura striata domestica*) studied here, adult males can sing but females cannot (Catchpole and Slater, 2008; Mooney et al., 2008), but recent studies have highlighted that female song is more common than was previously appreciated (Odom et al., 2014; Langmore, 2020). Females recognize individual males by their songs and evaluate the quality of those songs to choose one mate from many suitors (Catchpole, 1987; Searcy and Yasukawa, 1996; Nowicki and Searcy, 2004; Catchpole and Slater, 2008). Adult female songbirds can perceive and evaluate even very subtle differences between songs (Clayton, 1988; Searcy and Brenowitz, 1988; Cynx et al., 1990; Searcy, 1990; Vernaleo and Dooling, 2011), and they use the suite of information obtained from those song performances to select their mate (Catchpole and Slater, 2008).

Studies of songbirds provide a key experimental advantage in that song is a unimodal stimulus. Even though mate choice involves information obtained through a variety of modalities, song is so influential that females will solicit copulation by performing a copulation solicitation display (CSD) in response to song even if no male is physically present (Searcy and Marler, 1981; Searcy et al., 1981; Searcy and Andersson, 1986; Candolin, 2003; Riebel, 2009; Byers et al., 2010; Dunning et al., 2014). Female perception and mate choice are best measured through CSD production, but other behaviors may also serve as proxies of female mate preference. Prior work from our group revealed that a female BF’s song preference is reflected in not only the number of CSDs she performs but also the number of calls that she produces during song presentation (Dunning et al., 2014). Therefore, calls provide an easily detected and quantified measure of female mate preference (Dunning et al., 2014, 2020). In the present study, we use that well-established method to investigate the role of a specific set of auditory processing neurons in a female’s behavioral expression of mate choice.

Many previous studies have revealed specific auditory brain regions that are active in response to the playback of song (Woolley and Doupe, 2008; Prather, 2013; Monbureau et al., 2015; Van Ruijssevelt et al., 2018). The activity of those cells can be dependent on not only the acoustic properties of the stimulus but also more complex social contexts, such as whether the song was performed by a conspecific or a heterospecific, or whether the song was familiar, such as the song or call of a mate, vs. a novel song stimulus (Gentner et al., 2004; Terpstra et al., 2004). One brain region that exhibits such selective responses is the caudal nidopallium (NC). Previous studies of NC in male songbirds have shown that following lesions to the medial portion of this area (NCM), there is a reduction in song recognition and preference for familiar (tutor) song (Gobes and Bolhuis, 2007). Other studies have shown that when NCM or the central portion of NC (NCC) are temporarily inactivated, females show a decrease in affiliative behavior with conspecific males (Tomaszycki and Blaine, 2014; Van Ruijssevelt et al., 2018). Together these results point to NC as a site associated with complex perceptions, and they suggest that in female songbirds, NC plays a role in preference for songs performed by conspecifics. We extended that study to investigate the degree to which NC activity also shapes individual recognition of multiple conspecifics and selective preference for one individual among many suitors.

Here, we used a well-established means of measuring the preference of female Bengalese finches for one song among many conspecific stimuli (Dunning et al., 2014, 2020), and we combined that with an excitotoxic means of lesioning only somas within NC while leaving fibers of passage intact (Jarrard, 1989). We explored the role of NC in shaping female expression of behavioral indicators of mate preference by performing behavioral tests of mate preference before and after placement of bilateral lesions in NC of each subject. Our results reveal that chemically lesioning NC while leaving fibers of passage intact does not change a female’s ability or willingness to engage in behavioral indicators of mate preference, nor does it alter a female’s ability to identify the songs of individual males. Instead, lesion-induced changes in NC activity resulted in a change in the strength of preference for individual males. NC lesions result in a reduction of selectivity such that songs that were previously strongly preferred become less attractive, and songs that were previously least preferred become more attractive. Importantly, these reductions of selectivity do not result in a complete elimination of preference, indicating that activity in NC plays a key role in a female’s strength of preference for specific songs from among the array of song stimuli that she hears.

## Materials and Methods

### Care and handling of experimental subjects

We performed all experiments using adult (age >120 days post-hatch) BFs obtained from a commercial breeder (The Finch Farm, WA). All procedures were approved by the University of Wyoming Animal Care and Use Committee, and procedures were in compliance with recommendations from that group and state and federal regulations governing the housing and use of songbirds. A total of 28 (12 experimental, 10 controls, six shams) female subjects were used. At the end of behavioral recordings, birds were euthanized by an overdose of isoflurane and tissue was collected. Throughout all aspects of the study, care was taken to ensure health and comfort of all experimental subjects.

### Animal housing and preparation for recording

We maintained a 15:9 light:dark photoperiod, and seed, water and grit were available *ad libitum*. We housed females in groups of no more than six birds in a wire cage (41 cm × 33 cm × 24 cm) within a sound-attenuating chamber. Prior to experimentation, we identified males by song performance. Females were identified by the presence of calls but the absence of songs over the course of three or more days of continuous recording in a sound attenuating chamber (Dunning et al., 2014). Sex was confirmed histologically at the end of each experiment. Following well established protocols (Dunning et al., 2014, 2020), females were housed in all-female groups to prevent physical interaction with male birds, but females could still hear conspecific male songs from neighboring chambers. Females were housed in this all-female arrangement for 3–5 days prior to beginning behavioral tests. During that time, they did not interact with males of their species, but females could have heard faint vocalizations from conspecific males residing in other chambers in the lab (Vyas et al., 2009; Dunning et al., 2014). None of the females studied here had ever interacted with the males from whom the song stimuli were recorded. During behavioral tests, females were housed individually in sound-attenuating chambers where songs were played (see below) and the female’s behavioral responses (e.g., calls) were recorded by audio and video monitoring. At the time of each behavioral test, we moved the female to the chamber where we performed the behavioral tests (41 cm × 33 cm × 24 cm), and the bird was allowed to acclimate for at least 30 min after moving from the holding chamber and the beginning of testing (Banerjee and Adkins-Regan, 2011; Dunning et al., 2014, 2020).

### Song stimulus preparation and presentation

Similar to methods we have used previously to measure mate preference in female BF’s (Dunning et al., 2014), the undirected songs of six individual Bengalese finch males and one zebra finch male were recorded for at least 24 h (range 24–36 h) in a sound attenuated chamber in which we provided seed and water *ad libitum*. Because the males resided in the chamber alone, these were undirected song performances. We monitored vocal behavior using a microphone (Shure model SM57) positioned immediately adjacent to the bird’s cage (41 cm × 33 cm × 24 cm), and we used custom software to continually record sounds and save them onto a computer hard drive (Sound Analysis Pro; songs were bandpass filtered 300–10,000 Hz; Matlab software; Tchernichovski et al., 2000). For each male, we composed an aggregate song file from several individual song performances (average of five songs; range three to nine songs; individual songs were separated by 1 s of silence) which together were approximately 47 s (range 42–52 s) to control for song duration. Thus, each male was represented by a concatenated song stimulus containing several songs collected for a total duration of about 47 s of audio and silence (see Dunning et al., 2014 for more details). Importantly for the behavioral experimental design used here, there were individual-specific differences in the properties of these songs (e.g., different song properties for each male) but the same stimuli were used in behavioral testing of mate preference before and after placement of bilateral lesions in NC of each subject.

### Behavioral test of mate preference

Behavioral testing began immediately after the 30 min waiting period following transfer from the group housing cage to the individual testing cage (Dunning et al., 2014). Consistent with the female becoming acclimated during that time, females engaged in behaviors indicating their comfort (e.g., feeding, drinking, grooming), and in each test we ensured that we had observed these behaviors prior to beginning.

Song stimuli were presented at 70 dB through a speaker (Sound Acoustics) residing in the sound attenuated chamber (70 dB measured 13 cm from speaker, distance between the bird and the speaker varied from 5 to 22 cm based on the bird’s location as it moved within the cage). The sequence of song presentation was randomized for each test using a computer, with an interval of 20 s of silence between the aggregate song files of different males. We presented each aggregate song stimulus from each male once per test, and we tested birds no more than two times per day to prevent overexposure and habituation to the stimuli. We viewed the birds and recorded audio through a camera (General Electric model 45231) inside the sound attenuating chamber and measured each female’s behavioral responses in real time. Because the audio and video recording camera was so close to the female, we could detect even very low amplitude calls, and we could visually confirm the occurrence of a call. Females displayed many behaviors such as calling, perch hopping, beak swiping, and wing flapping when presented with the song stimuli. These behaviors have been noted previously, and calls have been shown to be closely related to the expression of CSDs and mate preference (Dunning et al., 2014). Therefore, we quantified mate preference by counting the number of calls that each female produced during playback of each song stimulus.

**Figure 1:**
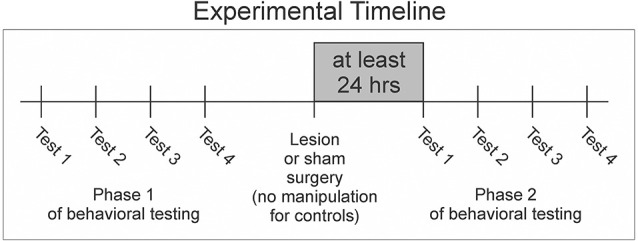
Females underwent two phases of behavioral testing. In each phase, females completed four valid tests of preference for songs performed by each of seven males. After the completion of Phase 1, experimental and sham birds received either lesion or sham surgeries before moving onto Phase 2. No manipulation was performed on control birds (detailed in “Methods” Section).

To ensure that we had sufficient resolution to define each female’s song preferences, we used well-established criteria to define a valid behavioral test of preference (Dunning et al., 2014). Each test involved playing all seven male song stimuli to each female. We required that female subjects called a minimum of 10 times in response to the song of at least one individual male or at least four times to the songs of at least two different males in order for a behavioral test to be considered valid (Dunning et al., 2014). If a bird did not complete four valid tests by the time that it had been tested eight times in Phase 1, then we ceased testing for that bird and excluded it from the Results (this was the case for only one of the females that we tested). We quantified the number of calls to six male BF conspecific song stimuli and one male zebra finch heterospecific song stimulus to determine each female bird’s rank ordering and strength of preference among song stimuli. All calls performed during song presentations were included in those computations. We performed behavioral tests of song preference before (Phase 1) and after surgical manipulation (Phase 2) to quantify any changes in preference that emerged as a result of lesioning NC (Figure 1).

### Surgery for placement of focal lesions

After recording the female’s pre-manipulation song preferences, we made bilateral chemical lesions to NC (*N* = 12 birds) using ibotenic acid (500 nl, 1 mg/ml in deionized water; Cayman Chemical, MN). During that procedure, birds were anesthetized with isoflurane (inhalation of 1%–3% in 100% oxygen) and placed in a customized stereotaxic device to immobilize the head and beak. We made a small incision in the scalp and created a small opening in the skull (head angle: 45°, 0.4 mm anterior, 2.5 mm lateral from mid sagittal sinus), and lowered a microsyringe (Neurosyringe, Hamilton company, MO) to a depth of 2.0 mm beyond contact with the surface of the brain. Following the injections, the exposed brain was covered with an inert polymer (Kwik-Sil, World Precision Instruments, FL), the skin was closed with a tissue adhesive (Vetbond, 3M, MN), and topical anesthetic was applied to the skin (4% lidocaine, HI-Tech, NY). Following surgery, all birds resumed their normal behavior within several hours.

Following complete recovery, each bird was returned to an individual-housing chamber and allowed to recover for at least 24 h before we retested song preferences in Phase 2. In our sham birds (*N* = 3 birds), the incision and opening in the skull were made, the injection syringe was inserted into the brain, but no drug was injected. In other sham birds (*N* = 3 birds), the incision and opening were made, we waited an amount of time comparable to that of the injection, and the opening and skin were resealed. In all measures reported in the Results, there were no significant differences between those groups. For example, three of the birds produced more calls after lesioning than before, and three produced fewer calls than before. Each of those trios contained members of each sham surgical treatment group, and on average there was no difference between the two sham surgical treatment groups. Therefore, both sham treatments are reported in the Results as sham birds (*N* = 6 birds). In our control birds (*N* = 10 birds), we made no surgical manipulation of any kind between phases one and two of behavioral testing. In all behavioral tests, we followed the same paradigm in Phase 2 (after lesioning CM or after performing sham surgery or in the second half of control testing) as we did in Phase 1 (prior to lesioning NC or prior to performing sham surgery or in the first half of control testing).

### Tissue collection and histological processing

After the end of post-manipulation behavioral testing, each female was deeply anesthetized using isoflurane and perfused transcardially with ice-cold 0.9% phosphate buffered saline followed by ice-cold 4% paraformaldehyde. We carefully removed the brain and placed it in 4% PFA for 24 h before being transferred to a 30% sucrose paraformaldehyde cryoprotecting solution for 72 h. We cut parasagittal sections (50 microns thickness) on a freezing microtome and placed them individually in wells containing phosphate buffer. Tissue sections were then mounted onto gelatin coated slides and allowed to dry overnight. The following day, tissue was stained with cresyl violet and viewed under a microscope (Olympus BX51 Brightfield Microscope; Olympus, PA) equipped with an RT-SE camera (SPOT 9.4 Slider-6, MA) and analyzed with SPOT software (version 5.1, MA). We compared our data to a stereotaxic atlas (Oregon Health and Science University, Portland, OR 97239^1^) to confirm accuracy of lesion placement. As elaborated in the Results, the extent of all lesions in birds described here was entirely within NC. Experimenters were blind to the results of the behavioral tests during the analysis of lesion placement.

### Statistical analysis

We quantified each female’s response to each song stimulus by counting the number of calls that the female performed in response to each stimulus (Dunning et al., 2014). We used those data to identify each female’s most-preferred through least-preferred stimulus. To compare the pre-manipulation vs. post-manipulation conditions, we used nonparametric tests to compare the number of calls produced, and we used two sample Kolmogorov-Smirnov tests to compare the distributions of responses across the seven males from which song stimuli were recorded (Sokal and Rohlf, 2011). We used linear regression to compute the slope and intercept of data used to quantify strength of preference, and those same tests enabled us to perform statistical comparisons of those slope and intercept values against expected models (e.g., is slope different than zero; Sokal and Rohlf, 2011). In all tests, significance was assessed at alpha = 0.05. Values are reported as either individual values (e.g., range of numbers of calls) or means ± SE.

## Results

### NC lesioned females were unchanged in their ability to indicate mate preference

**Figure 2:**
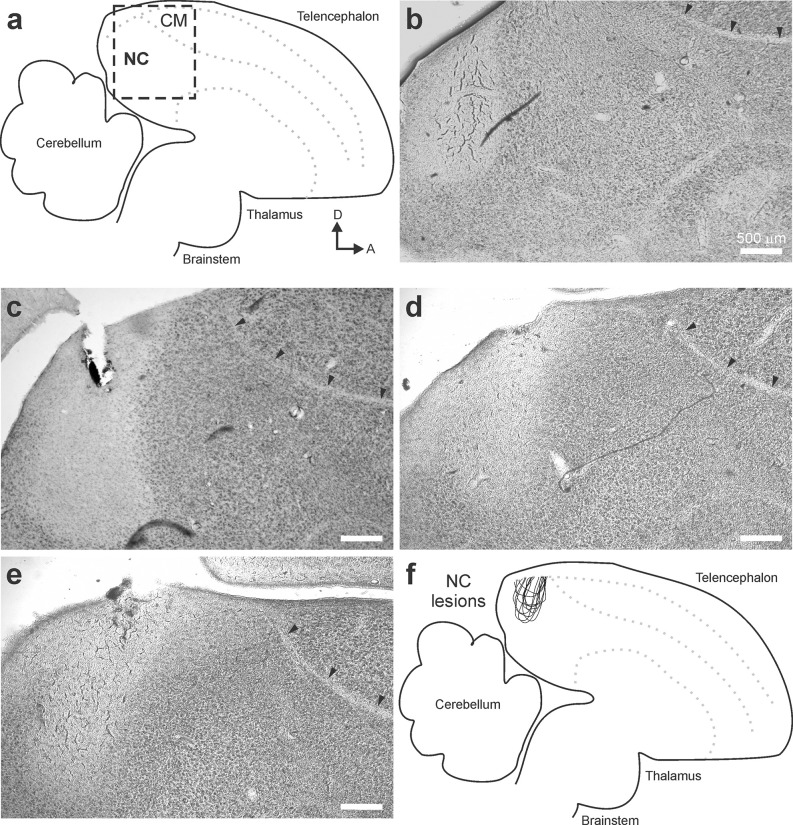
Lesions were restricted to NC. **(A)** The location of NC was recognized by its relative position in the brain as well as lamina that serve as anatomical landmarks. **(B–E)** At the end of each experiment, brain tissue was collected and used to verify the location of each lesion in each hemisphere. In no case did lesions affect tissue anterior of the lamina mesopallialis (small arrows; an easily recognizable interface between NC and CM) or inferior of the dorsal arcopallium lamina (not shown; another easily recognizable interface between the nidopallium and the arcopallium). **(F)** An overlay of traces of individual lesion sites confirmed that all lesions were restricted to NC.

**Figure 3:**
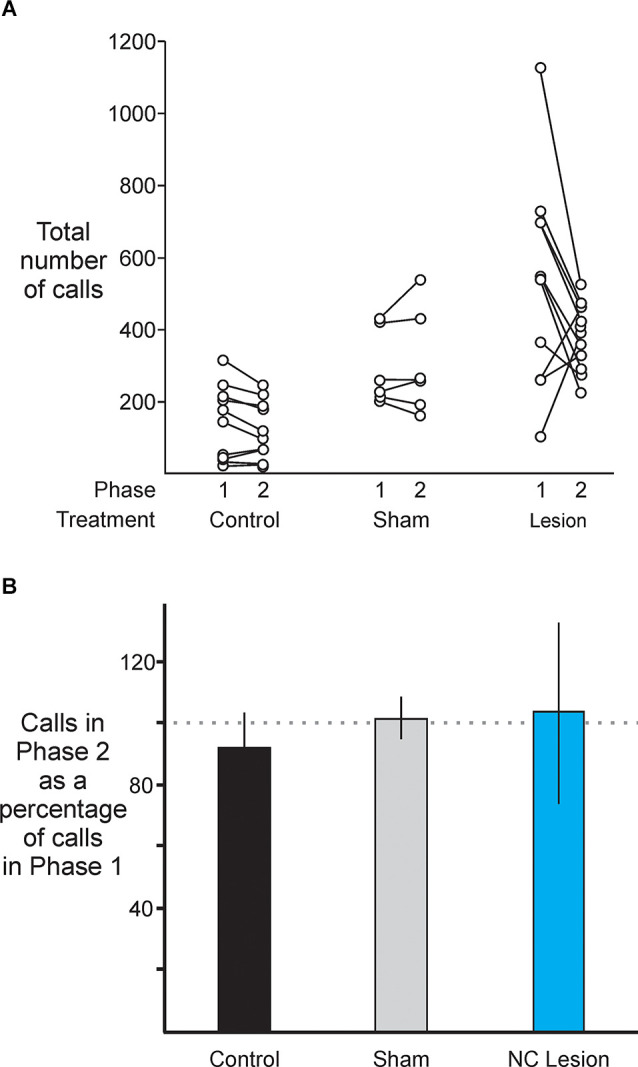
NC lesioned females were unchanged in their ability to indicate mate preference. **(A)** Females typically responded to song stimuli by producing large numbers of calls, and the numbers of calls produced varied across birds (individual data points connected by a line indicate the responses of an individual female in Phase 1 and Phase 2). **(B)** Experimental birds (blue bar, right; *N* = 11) expressed no significant change in their number of calls performed in Phase 2 as a percent of calls performed in Phase 1 of behavioral testing (bars and lines indicate means and SE; paired t-test, *t* = 0.50; *p* = 0.62). No change between Phase 1 and Phase 2 in the number of calls produced in response to all song stimuli was also evident for control (black bar, left; paired t-test, *t* = 1.01, *p* = 0.33, *N* = 10 birds) and sham conditions (gray bar, middle; paired t-test, *t* = 0.20, *p* = 0.85, *N* = 6 birds).

All lesions were restricted to NC (Figure 2, *N* = 12 birds). Lesions commonly extended along the axis on which the injection was made (Figures 2B–E). Similar to results from previous injections in NC, affected volumes typically extended 250–350 microns in the medial-lateral direction (Bloomston et al., 2022). As in previous reports, song playback resulted in call responses during the song presentation (Dunning et al., 2014, 2020). Calls produced by the females in this study were trills of the type described previously as amplitude modulated calls in adult female Bengalese finches (Yoneda and Okanoya, 1991). On average, female birds called 302 ± 29 times in response to all song stimuli in Phase 1 or Phase 2 (range 16–1,127; Figure 3A). These numbers of calls provided sufficient quantity and resolution to compare treatment groups.

To investigate the possibility that lesions in NC could induce a change in the bird’s ability to produce vocal indicators of mate preference (calls), we compared the number of calls that each female produced in response to all song stimuli before and after lesioning NC. In our experimental birds (*N* = 12 birds, blue bar on the right in Figure 3B), we observed no significant change in the number of calls evoked by male song stimuli before (Phase 1) and after lesioning (Phase 2; signed rank test, *p* = 0.41). No change from the first to second phase of behavioral testing was also evident for control birds (black bar on the left in Figure 3B; signed rank test, *p* = 0.38, *N* = 10 birds) and birds that received sham surgeries but had no lesions placed (gray bar in the middle in Figure 3B; signed rank test, *p* = 0.69, *N* = 6 birds). Together, these results indicate that lesioning NC had no significant effect on females’ ability or willingness to produce calls in response to male song.

### NC lesioned females were unchanged in their ability to identify songs of individual males

**Figure 4:**
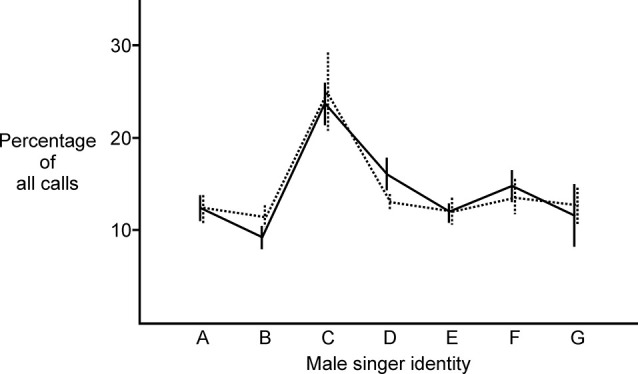
NC lesioned females were still able to identify the songs of individual males. After lesioning NC, there was no significant change in the distribution of their song preferences for specific males (two sample Kolmogorov-Smirnov test, KS statistic = 0.08, *p* = 0.92, *N* = 11 birds; solid line indicates the average percentage of calls for experimental birds in Phase 1; dotted line indicates the average percentage of calls for the same set of experimental birds in Phase 2; heterospecific song is represented by letter G). This absence of change between the first and second behavioral tests was also evident for control birds and sham birds (detailed in “Results” Section).

Lesions to NC also had no significant effect on female birds’ ability to identify the songs of individual males. Comparing pre-lesion responses (solid line in Figure 4) to post-lesion responses (dotted line in Figure 4), females exhibited no significant change in the distribution of their song preferences for specific males (two sample Kolmogorov-Smirnov test, KS statistic = 0.08, *p* = 0.92, *N* = 12 birds). This absence of change between the first and second behavioral tests was also evident for control birds (two sample Kolmogorov-Smirnov test, KS statistic = 0.10, *p* = 0.80, *N* = 10 birds) and sham birds (two sample Kolmogorov-Smirnov test, KS statistic = 0.17, *p* = 0.56, *N* = 6 birds). Taken together, these results indicate that not only were females still able to hear after lesions in this auditory area, they were also still able to identify the songs of individual males.

The conspecific song represented by letter C was generally more attractive to females than songs from other males. This pattern of one song being more broadly attractive than others has been reported previously along with an exploration of features that distinguish attractive songs from others (Dunning et al., 2020). From these data, it appears that activity in the portion of NC that we lesioned in these experiments does not play an important role in a female’s ability to identify individual males based on song. As reported for many species in many studies, females generally found conspecific song more attractive than heterospecific song (reviewed in Catchpole and Slater, 2008). Prior to lesioning, 9 of 12 females found the average conspecific song more attractive than the single heterospecific stimulus, and that pattern was present in 8 of 12 females after lesioning (changes in song preference after lesioning are considered in greater detail below).

### NC lesioned females were unchanged in their rank ordering of song preference

**Figure 5:**
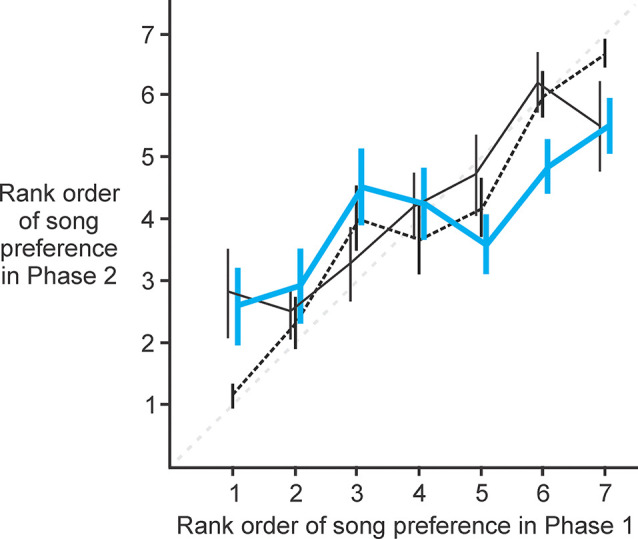
NC lesioned females were unchanged in their rank ordering of song preferences. Data from experimental (thick blue solid line; paired t-test, *t* = 0.10, *p* = 0.92), control (thin black solid line; paired t-test, *t* = 0.48, *p* = 0.48), and sham birds (thin black dashed line; paired t-test, *t* = 0.00, *p* = 1.00) all revealed no significant change in rank ordering of preference from Phase 1 to Phase 2 of behavioral testing (thin gray dashed line indicates the line of identity representing no change in rank ordering from Phase 1 to Phase 2).

To determine if activity in NC is related to the female’s ranking of preference for individual males, we analyzed each female’s rank ordering of the seven male songs before and after lesions to NC (Figure 5). We compared the rank that each female assigned to each male prior to lesioning (Phase 1) vs. the rank that she assigned to that same male after lesioning (Phase 2). In this paradigm, no change in rank ordering of preference would be evident as data points lying along the line of identity (thin gray dashed line in Figure 5). Data from experimental (thick blue solid line in Figure 5; paired t-test, *t* = 0.10, *p* = 0.92), control (thin black solid line in Figure 5; paired t-test, *t* = 0.48, *p* = 0.48) and sham birds (thin black dashed line in Figure 5; paired t-test, *t* = 0.02, *p* = 0.99) all revealed no significant change in rank ordering of preference from the first to the second behavioral test. Thus, we detected no significant change in the rank ordering of the females’ most- to least-preferred songs, indicating that NC does not play an important role in the rank ordering of song preferences.

### NC lesioned females had a reduction in choosiness for male song

**Figure 6:**
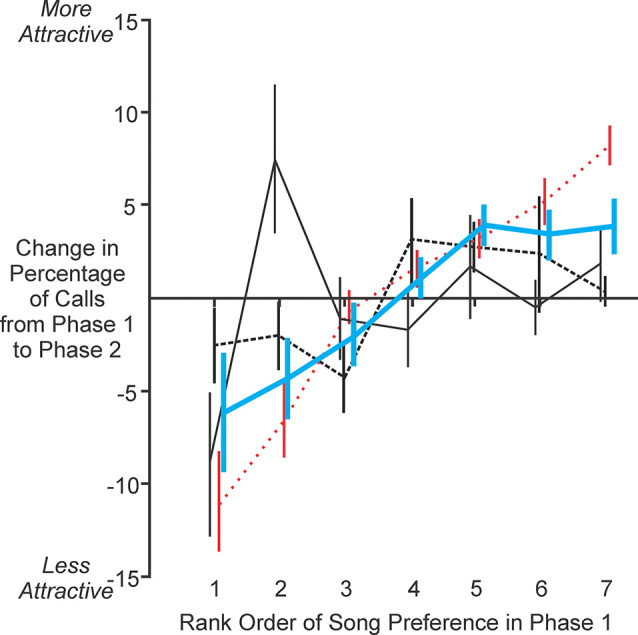
NC lesioned females expressed reduced strength of preference among songs of different males. For experimental birds (thick blue solid line), data were arranged in a linear manner with significantly positive slope (linear regression, *F* = 28.14, slope = 1.84, *p* < 0.001), indicating that lesioning NC caused birds to become less choosy for songs that they previously found attractive or unattractive (detailed in “Results” Section). In contrast, data from control birds (thin black solid line; linear regression, *F* = 1.43, *p* = 0.24) and sham birds (thin black dashed line; linear regression, *F* = 1.30, *p* = 0.26) had slopes that were not significantly different than zero, indicating that when NC was left unaltered, females expressed no change in choosiness for attractive vs. unattractive songs. We created a model (thin red dotted line) to simulate a scenario in which NC lesions caused preferences to become completely indistinguishable across all stimuli (i.e., one seventh of all calls produced in response to each of the seven stimuli). Data from that model yielded a slope that was significantly greater than zero (linear regression, *F* = 113.22, slope = 3.05, *p* < 0.001) and significantly steeper than the slope of 1.84 observed for our experimental data (linear regression of experimental data minus model data, *F* = 21.00, *p* < 0.001). Thus, NC lesions induced changes in preference but not a complete loss of preference, suggesting that NC plays a role in establishing a female’s strength of preference among different songs.

To investigate the degree to which activity in NC may be associated with the strength of song preference, we analyzed females’ changes in preference for each song following lesioning of NC. To quantify that parameter, we computed the difference in the percentage of all calls that was produced in response to each song stimulus in Phase 2 compared to the response to the same song in Phase 1 (Figure 6). Using this measurement, a positive change would indicate that the bird expressed a greater preference for that song (i.e., found it more attractive) following NC lesion, a negative change would indicate that the bird expressed a lesser preference for that song (i.e., found it less attractive) after lesioning, and no change would indicate that the bird’s preference was the same in the first and second tests of song preference. Therefore, viewed in the way that the data are presented in Figure 6, a positive slope would indicate that the bird became less choosy for attractive vs. unattractive songs following lesion, a negative slope would indicate that the bird became more choosy, and a slope of zero would indicate no change in choosiness for songs that the bird found attractive vs. unattractive.

For experimental birds, the data were arranged in a linear manner with a slope that was slightly but significantly greater than zero (thick blue solid line in Figure 6; linear regression, *F* = 28.14, slope = 1.84, *p* < 0.001), indicating that lesioning NC caused birds to become less choosy for songs that they previously found attractive or unattractive (Figure 6). In contrast, data from control birds (thin black solid line in Figure 6; linear regression, *F* = 1.43, *p* = 0.24) and sham birds (thin black dashed line in Figure 6; linear regression, *F* = 1.30, *p* = 0.26) had slopes that were not significantly different than zero, indicating that females expressed no change in choosiness for attractive vs. unattractive songs when NC was left unchanged. These data reveal that birds express reduced choosiness in their mate preference following lesions of NC.

To investigate the possibility that NC lesions might have eliminated choosiness altogether, we created a model to simulate the scenario in which preferences were the values that we measured in the first behavioral test of preference and then became entirely uniform (went to chance) for each of the seven song stimuli in the second test (i.e., 14.3% of all calls produced in response to each stimulus). Data from that model yielded a slope that was significantly greater than zero (thin red dotted line in Figure 6; linear regression, *F* = 113.22, slope = 3.05, *p* < 0.001) and significantly steeper than the slope of 1.84 observed for our experimental data (linear regression of experimental data minus model data, *F* = 21.00, *p* < 0.001). Therefore, our experimental data for lesions of NC are intermediate between a model of no change in choosiness for attractive vs. unattractive songs (i.e., a slope of zero along the x-axis in Figure 6) and a model of complete elimination of choosiness (i.e., the red model data in Figure 6). Together, these results indicate that lesioning NC caused a reduction but not complete elimination of females’ choosiness for songs that they found attractive or unattractive prior to lesioning.

## Discussion

These experiments revealed that chemically lesioning NC does not change a female’s ability to perform calls. Females are also unchanged in their overall amount of call production following NC lesions, suggesting that they were also unchanged in their willingness to produce calls. It is possible that volition is affected one way by an NC lesion and general arousal is affected in another way that resulted in an overall unchanged amount of call output, but that possibility could not be addressed here. Future studies using methods such as a forced choice paradigm could yield further insight. In addition, lesioning NC does not change the bird’s ability to identify the songs of individual males, nor does it alter the rank ordering of the female’s preferences. However, lesioning NC does result in a statistically significant decrease in the strength of selectivity for a female’s song preferences. Importantly, that decrease does not result in a complete elimination of preference, as female preferences are still evident but are not as strongly expressed after lesioning NC. Taken together, these data suggest that NC plays a role in a female’s strength of preference (“choosiness”) in song evaluation and mate choice.

A bird that was originally very choosy for a particular song stimulus typically became less choosy after NC was lesioned, which was evident as a reduction in the percentage of calls performed in response to that song stimulus. Additionally, a stimulus that was a female’s least preferred prior to lesioning typically became more attractive (or less unattractive) after NC lesioning. Thus, NC appears to play a role in establishing the degree to which a female bird is selective in the magnitude of her responses to songs of different males. This is especially evident in the case of a female’s choosiness for her most- and least-preferred song stimuli. The most attractive song became less attractive, and the least attractive (or perhaps even unattractive) song elicited more responses. These results suggest that activity in NC is closely related to the degree to which females are able to disambiguate male songs according to their subjective value.

We have previously described features of male BF song that influence female evaluation of its attractiveness (Dunning et al., 2020). A goal of future studies could be to extend those and other related studies by comparing the roles of specific brain sites in evaluating the quality of not only undirected vs. directed song performances but also familiar vs. unfamiliar and conspecific vs. heterospecific songs with a range of similarity to the songs of the female’s own species. We have noted here that the calls produced by these females were of the type described as amplitude modulated calls in female Bengalese fiches (Yoneda and Okanoya, 1991). These are trills of different duration (typically 2–4 notes). Each type is performed in response to a song that a female finds attractive (Dunning et al., 2014). Additional future work could investigate the degree to which subtle differences in a female’s evaluation may be evident in the degree to which she produces different proportions of different call types.

Our data reveal a role for NC in female mate preference, but NC is not alone in this functionality. It is reasonable to posit that the neural circuits underlying processes as complex as song perception and subsequent mate choice include additional selectively responsive auditory sites such as the caudal mesopallium (CM), the nucleus interfacialis (NIF), HVC shelf, and Field L because of their selective responses to auditory stimuli (Mello and Clayton, 1994; Gentner et al., 2001; Keller and Hahnloser, 2009). Among these sites, CM has been implicated in shaping female responses to songs of their own species such that birds are more responsive to heterospecific song following lesions to CM (Macdougall-Shackleton S. et al., 1998). CM is a particularly attractive candidate to shape selective expression of behavioral indicators of mate choice because its efferent pathways link sensory information to areas involved with motor production of courtship behaviors such as calls (Dunning et al., 2018).

Previous work has shown that NC is reciprocally interconnected with cells in CM (Vates et al., 1996; Dunning et al., 2018; Bloomston et al., 2022), suggesting that NC and CM may work together to control over courtship behaviors. In support of the idea that modulation of song evaluation and courtship behavior may require the coordination of activity in both of those sites, roughly half of the neurons within NC are GABAergic with projections to CM (Pinaud et al., 2008). Therefore, at least a portion of NC neurons provide inhibitory tone onto CM (Pinaud et al., 2008). Through that network, a lesion-induced decrease in inhibition between NC and CM could make CM neurons more responsive to auditory stimuli. In turn, this disinhibition could result in increased activity in sites to which CM projects. In this scenario, lesioning NC could result in disinhibition of CM projection neurons, and that could account for the observed increase in courtship behaviors in response to least preferred songs.

It could also be the case that lesioning NC could result in disinhibition of GABAergic interneurons in CM, causing less activity in CM projection neurons and resulting in lesser production of female courtship behaviors in response to songs. Within CM and NC, neurons are selective for natural vocalizations, and that selectivity may help females to assign identity to the vocalizations of specific individuals, such as songs or calls that distinguish the male that the female finds most attractive (Menardy et al., 2012). Furthermore, these neurons’ response strength is different for familiar male vs. unfamiliar male and female calls (Giret et al., 2015). This level of selectivity further suggests that these neurons may contribute to recognizing the identity of the source of a vocal signal. This idea gives rise to the testable hypothesis that songs with different degrees of subjective value drive activity in different classes of neurons (projection neurons vs. interneurons). An important goal of future experiments will be to use very precise stimulation methods to test that idea, such as optogenetic stimulation of specific subpopulations of neurons in NC and CM (Elie et al., 2019).

Two subregions of NC have been shown to play important roles that could contribute to song evaluation and mate choice. A subregion in the medial and caudal portion of NC (caudomedial nidopallium; NCM) has been associated with female preference (Tomaszycki and Blaine, 2014), and a subregion in the central and caudal portion of NC (caudocentral nidopallium; NCC) has been postulated as a center for memorization and integration of auditory experiences that impact mate preference (Van Ruijssevelt et al., 2018). Future investigations should focus on categorizing different subregions of NC. This could be done using molecular tools to identify the types of cells present and their relative prevalence across different regions of NC. Additional studies could use focal lesions and electrophysiological recordings in awake and freely behaving birds to reveal the specific roles that activity in these subregions may have in the processing of male song and production of call responses. Tract-tracing studies using precise approaches to focally apply tracer molecules or label specific cells using viral transfection should also be performed to more completely describe the circuits through which NC may play a role in processing sensory information and using that activity to direct specific motor outcomes (Bloomston et al., 2022).

Results from our group have shown that injections of anterograde tracer molecules into NC reveals clusters of axons and varicosities in the ventral portion of the intermediate arcopallium (AIV; Bloomston et al., 2022). AIV in male songbirds has been implicated in perceiving and learning vocalizations, serving as a driver of motivational state and reinforcement learning through its connections to the ventral tegmental area (VTA) and the substantia nigra pars compacta (SNc; Mandelblat-Cerf et al., 2014). Interestingly, a similar projection has been observed following anterograde tracer injections into CM (Dunning et al., 2018). Future studies of the neural basis of female mate choice should investigate the importance of this convergence from the auditory processing areas NC and CM onto AIV and this dopaminergic pathway implicated in behavioral motivation and reward. It is enticing to suspect that this network may influence female song evaluation and production of behavioral responses such as calls and CSDs.

The present results reveal that NC helps to shape female evaluation of the quality of the songs performed by different males. The significance of these findings is amplified by other results implicating CM as also playing a similar role (Macdougall-Shackleton S. A. et al., 1998) and tracing studies that reveal how activity in NC and CM can influence activation of downstream pathways implicated in motivation and production of courtship behaviors (Vates et al., 1996; Dunning et al., 2018; Bloomston et al., 2022). The link between sensory perception and selective motor activation lies at the heart of decision making, and the present results point to NC as a contributor in that process for song evaluation and mate preference. Continued study combining optogenetic, electrophysiological, and behavioral approaches will be essential to discern the degree to which activity in NC, CM, and specific downstream pathways contribute to evaluation of sensory perception, production of motor indicators of mate choice, or both. Future studies can also target different subregions of NC and use behavioral tests in addition to those used here. For example, a second means of assessing a female’s song preference could be used to disambiguate a change in a female’s evaluation of song quality vs. a change in her ability or desire to produce the behavioral indicator of mate preference studied here (calls). Very precisely targeting different subregions of NC (e.g., NCC, NCM, NCL) could reveal whether circuits in those subregions may contribute different facets of song evaluation and mate preference. A long term goal should be to couple these techniques with a thorough knowledge of the female’s life experience. Females differ in what song they find most attractive (e.g., Dunning et al., 2014), and a female’s song preference and activity in auditory processing areas such as NC and CM can be affected by experience (reviewed in Fujii et al., 2022). Coupling detailed knowledge of a female’s life experience (e.g., reared in the laboratory under known acoustic and social conditions) with awake recording or optogenetic stimulation of activity in these auditory areas and their downstream targets holds the promise of revealing new insights into how this system processes sensory information in service of behavioral activation. Such studies would enable researchers to harness individual variation as a means of discovering the features that sculpt the neural basis of song evaluation and mate choice. With the present results and the insights that will emerge from those future experiments, the songbird model will continue to emerge as an especially tractable means of identifying the neural mechanisms of decision-making.

## Data Availability Statement

Relevant data are available from the Open Science Framework at: https://osf.io/54gr8/.

## Ethics Statement

The animal study was reviewed and approved by the University of Wyoming Animal Care and Use Committee, and procedures were in compliance with recommendations from that group and state and federal regulations governing the housing and use of songbirds.

## Author Contributions

KL and JP designed, planned, oversaw the study, and wrote the first draft of the manuscript. KL, TF, EP, HH, KZ, and PS contributed to the data collection. KL, AC, and JP contributed to the data analysis. All authors provided comments on the work. All authors contributed to the article and approved the submitted version.

## Funding

This work was supported by the National Science Foundation (NSF IOS CAREER 1453084 to JP). Students were supported by Wyoming NIH INBRE (NIH# 2P20GM103432; grants to TF, EP, HH, and KZ) and an NSF GRFP awarded to KL. Funding sources had no involvement in study design or any aspect of manuscript preparation.

## Footnotes

^1^ http://www.zebrafinchatlas.org
